# Retrospective Analysis of HLA Class II-Restricted Neoantigen Peptide-Pulsed Dendritic Cell Vaccine for Breast Cancer

**DOI:** 10.3390/cancers16244204

**Published:** 2024-12-17

**Authors:** Takafumi Morisaki, Makoto Kubo, Shinji Morisaki, Masayo Umebayashi, Hiroto Tanaka, Norihiro Koya, Shinichiro Nakagawa, Kenta Tsujimura, Sachiko Yoshimura, Kazuma Kiyotani, Yusuke Nakamura, Masafumi Nakamura, Takashi Morisaki

**Affiliations:** 1 Department of Surgery and Oncology, Graduate School of Medical Sciences, Kyushu University, Fukuoka 812-8582, Japan; smiley199903@yahoo.co.jp (T.M.); kubo.makoto.804@m.kyushu-u.ac.jp (M.K.); nakamura.masafumi.861@m.kyushu-u.ac.jp (M.N.); 2 Department of Cancer Immunotherapy, Fukuoka General Cancer Clinic, Fukuoka 812-0018, Japan; shinji.m.03235@gmail.com (S.M.); atagoul-@hotmail.co.jp (M.U.); htanaka@cancer-clinic.jp (H.T.); fgcckoya@gmail.com (N.K.); nakagawaes@yahoo.co.jp (S.N.); fgcc.tsujimura@gmail.com (K.T.); 3 Department of Medicine and Clinical Science, Graduate School of Medical Sciences, Kyushu University, Fukuoka 812-8582, Japan; 4 Cancer Precision Medicine Inc., Kawasaki 213-0012, Japan; yoshimura@cancerprecision.co.jp; 5 National Institutes of Biomedical Innovation, Health and Nutrition, Osaka 567-0085, Japan; k-kiyotani@nibiohn.go.jp (K.K.); yusuke-nakamura@nibiohn.go.jp (Y.N.)

**Keywords:** neoantigen, HLA class II, dendritic cell, vaccine, breast cancer

## Abstract

**Background/Objectives**: Neoantigens have attracted attention as ideal therapeutic targets for anti-tumour immunotherapy because the T cells that respond to neoantigens are not affected by central immune tolerance. Recent findings have revealed that the activation of CD4-positive T cells plays a central role in antitumor immunity, and thus targeting human leukocyte antigen (HLA) class II-restricted neoantigens, which are targets of CD4-positive T cells, is of significance. However, there are very few detailed reports of neoantigen vaccine therapies that use an HLA class II-restricted long peptide. In the present study, we retrospectively analysed the ability of HLA class II-restricted neoantigen-pulsed dendritic cell vaccines to induce immune response in five breast cancer patients. **Methods**: We performed whole exome and RNA sequencing of breast cancer tissues and neoantigen prediction using an in silico pipeline. We then administered dendritic cells pulsed with synthesized an HLA class II-restricted long peptide containing an epitope with high affinity to HLA class I in the lymph node. **Results**: ELISPOT analysis confirmed that a T-cell response specific for the HLA class II-restricted neoantigen was induced in all cases. TCR repertoire analysis of peripheral blood mononuclear cells before and after treatment in three patients showed increases of specific T-cell clones in two of the three patients. Importantly, no recurrence was observed in all patients. **Conclusions:** Our analysis demonstrated the immunological efficacy of the HLA class II-restricted neoantigen peptide dendritic cell vaccine against breast cancer and provides useful information for the development of neoantigen vaccine therapy for breast cancer.

## Simple Summary

Cancer vaccines targeting neoantigens represent a new modality for cancer treatment. We used a neoantigen prediction pipeline involving a combination of whole genome and RNA sequencing and in silico analyses to predict neoantigens and pulsed patient-derived dendritic cells with the synthesized HLA class II peptides. We administered this vaccine (Neo-P DC vaccine) to five breast cancer patients post-surgery to evaluate the immune response against the Neo-P Dc vaccine. We confirmed a lymphocyte response particularly for HLA class II-restricted neoantigens containing HLA class I epitopes in all cases. No relapses have been reported. These results indicate the immunological efficacy of the HLA class II-restricted neoantigen peptide dendritic cell vaccine against breast cancer.

## 1. Introduction

Neoantigens are tumour-specific antigens that arise from genetic mutations that result in changes in amino acid sequences. Because neoantigens are HLA-restricted peptides that are not expressed in normal cells, the T cells that react to neoantigens may escape central tolerance [1,2,3]. When neoantigen-reactive T cells are activated via dendritic cells, they recognise tumour cells that present neoantigens on the cell surface together with HLA class I as non-self and can exert potent tumour-specific cytotoxic activity. Neoantigen-specific T cells play an important role in the effectiveness of immune checkpoint inhibitors, which have been shown to be effective in various types of cancer in recent years [4,5,6,7,8].

Until recently, cancer vaccine therapy has focused on shared antigens that are also expressed in normal cells, such as tumour tissue overexpressed antigens (HER2, CEA) and cancer testis antigens (NY-ESO-1, Mage-3) [9]. However, these vaccines have not demonstrated significant effects because of their low ability to induce immunity. Advances in genetic analysis technology using next generation sequencing and artificial intelligence have made it possible to predict HLA class I-restricted neoantigens, and the development of vaccine therapies targeting neoantigens has progressed rapidly, with their safety and efficacy demonstrated in clinical trials [10,11,12,13]. In addition to HLA class I-restricted neoantigens, the importance of HLA class II-restricted neoantigens is becoming clear [14,15,16].

Among the various cancer vaccine therapies, dendritic cell vaccines have been demonstrated to induce T-cell immune responses and exhibit safety [17,18]. In Japan, dendritic cell vaccine therapy is now being performed as a treatment for various cancers as a result of the regenerative medicine law. We have been performing neoantigen peptide-pulsed dendritic cell vaccine therapy for various solid cancers using a neoantigen prediction pipeline based on in silico analysis since 2019 and have reported on its safety and efficacy [19,20].

Breast cancer is the most common cancer in women, and the number of cases continues to increase [21]. Most breast cancers are detected at an early stage, and the risk of recurrence can be reduced through surgery and drug therapy to prevent recurrence, but there are still many cases of recurrence [22]. In cases of recurrence, long-term chemotherapy is required, and it is difficult to achieve a cure. Therefore, effective strategies to prevent breast cancer recurrence are urgently needed.

We have started administering HLA class I+II affinity neoantigen-pulsed dendritic cell vaccines as a means of preventing recurrence after surgery for breast cancer. In this study, we retrospectively analysed the ability of our established neoantigen-pulsed DC (Neo-P DC) vaccine to induce an immune response in breast cancer patients. We selected long peptides with HLA class II affinity that include HLA class I-restricted neoantigen epitopes, and the induction of T-cell immune responses was confirmed in all cases using ELISPOT analysis. Our results demonstrate the immunological effects of HLA class II-restricted neoantigen peptide-pulsed dendritic cell vaccines for breast cancer. We believe these findings will provide useful information for the development of vaccine therapy for breast cancer.

## 2. Materials and Methods

This study adhered to the ethical guidelines set forth by the Ethical Committees of Kyushu University Hospital (Approval No. 128) and the Fukuoka General Cancer Clinic (Approval No. FGCC-EC009). All procedures were conducted in compliance with the Act on Securement of Safety on Regenerative Medicine in Japan and aligned with the principles of the Declaration of Helsinki. Written informed consent was obtained from all patients.

### 2.1. Breast Cancer Patients and Samples

This study included five patients with histologically confirmed breast cancer from Kyushu University and Fukuoka General Cancer Clinic. The clinical characteristics are shown in Table 1. All patients underwent curative surgery for breast cancer before the study. Additional detailed pathological information is provided in Supplementary Table S1.

Breast cancer specimens were collected through tumour resection. Peripheral blood mononuclear cells (PBMCs) were isolated via leukapheresis using the Haemonetics Component Collection System (Haemonetics, Boston, MA, USA), following the manufacturer’s guidelines. The leukapheresis product was diluted with RPMI-1640 medium (Kojin-Bio Inc., Saitama, Japan) and subjected to density gradient isolation using Ficoll-Paque. The isolated cells were then washed three times with RPMI-1640 medium and cryopreserved at −80 °C. Tumour specimens were collected immediately prior to neoantigen prediction and dendritic cell vaccine therapy.

### 2.2. Whole Exome Sequencing and RNA Sequencing (RNA-seq)

Genomic DNA and total RNA were isolated from formalin-fixed paraffin-embedded tissues using the AllPrep DNA/RNA Mini Kit (Qiagen Inc., Venlo, The Netherlands), following the manufacturer’s protocol. Control genomic DNA was obtained from patient-matched PBMCs. Whole exome libraries were generated from 200–3000 ng genomic DNA using the SureSelect Human All Exon V6 kit (Agilent Technologies Inc., Santa Clara, CA, USA, adhering to the manufacturer’s guidelines. RNA-seq libraries were prepared with the TruSeq Stranded mRNA Library Prep Kit (Illumina Inc., San Diego, CA, USA). Sequencing of the whole exome and RNA-seq libraries was performed using a HiSeq 4000 (2 × 100 bp) or NovaSeq 6000 (2 × 150 bp) sequencer (Illumina).

### 2.3. Read Mapping and Variant Calling

For whole-exome sequencing, sequence reads were aligned to the human reference genome (GRCh37/hg19) using the Burrows–Wheeler Aligner (v0.7.10) [23]. Possible PCR duplicates, read pairs with a mapping quality score below 30, and reads with more than 5% mismatches were excluded. For RNA sequencing (RNA-seq), reads were mapped to the GRCh37/hg19 genome using STAR (v2.4.0a) [24]. Somatic variants were identified using Fisher’s exact test-based methods with the following parameters, as previously described [19]: (i) base quality ≥ 15, (ii) sequencing depth ≥ 10, (iii) variant depth ≥ 4, (iv) variant frequency in the tumour ≥ 10%, (v) variant frequency in normal samples < 2%, and (vi) Fisher *p*-value < 0.05. Single nucleotide variants (SNVs) were annotated using ANNOVAR [25].

RNA-seq data from tumours were analysed to assess gene expression. The read counts covering somatic mutation sites were used to confirm the mRNA expression of mutated genes [19]. Each RNA-seq dataset contained more than 20 million total reads per sample.

### 2.4. Neoantigen Prediction

Neoantigen analysis was conducted as previously described [19]. In brief, HLA class I genotypes for the patients were inferred from whole-exome sequencing data of peripheral blood using the OptiType tool v1.3.5 [26]. HLA class II genotypes were determined with the PHLAT tool [27]. Neoantigens were predicted for each non-synonymous variant, and the binding affinities of short peptides (8- to 11-mer) for HLA-A, HLA-B, and HLA-C were assessed using the computational tools NetMHC v3.4 and NetMHCpanv2.8, as described previously [19]. The binding affinities of long peptides (15- to 18-mer) for HLA-DRB1 were analysed using netMHCII-2.2 and netMHCII-pan-3.1 [28]. Neoantigen peptides were defined as those with predicted binding affinities to HLA-A, HLA-B, and HLA-C of IC50 < 500 nM or HLA-DRB1 < 500 nM. The number of mutations and predicted neoantigens in patients is shown in Table 2.

Neoantigen peptides with a predicted binding affinity of ≤50 nM, based on the concentration required for half-maximal inhibition, were chosen for further analysis. mRNA expression was combined to identify potential neoantigen candidates. Peptides were synthesized, and their quality was verified using high-performance liquid chromatography. For the vaccine selection, we focused on HLA class II-restricted neoantigen long peptides, taking into account not only their high affinity and the mRNA expression levels of mutated genes, but also their potential to contain amino acid sequences with strong binding affinity to HLA class I, namely HLA class I-restricted neoantigens. The list of selected neoantigens for each case is provided in Table 3.

### 2.5. Generation and Administration of DC Vaccines

DC vaccines were prepared following previously established methods [20,29]. In brief, PBMCs, obtained via pretreatment leukapheresis, were thawed and seeded into six-well plates containing complete medium with 1% autologous serum (2 × 10⁶ cells/well in 2 mL of medium). After a 30 min incubation, non-adherent cells were removed, and the plates were washed with RPMI. The remaining adherent cells were cultured in DC-specific medium supplemented with 100 ng/mL granulocyte-macrophage colony-stimulating factor (Primmune Inc., Kobe, Japan) and 50 ng/mL interleukin 4 (Primmune Inc.).

On day six, the culture was supplemented with two maturation factors: 500 IU/mL tumour necrosis factor-α (PeproTech Inc., Cranbury, NJ, USA) and 500 IU/mL interferon-α (Sumitomo Pharma, Osaka, Japan). Morphological changes were observed via light microscopy, and cells were characterized through flow cytometric analysis. Mature DCs (mDCs) were defined by high expression levels of HLA class I, HLA-DR, CD40, and CD86, alongside a lack of CD14 expression.

Neoantigen peptides were synthesized, dissolved in sterile water with dimethyl sulfoxide, filtered through a 0.22 µm syringe (Millipore, Molsheim, France), and tested for endotoxin, β-glucan, and mycoplasma, all of which were below detectable levels. Endotoxin and β-glucan levels were measured using a Toxinometer ET-6000 (Wako Pure Chemical Industries, Ltd., Osaka, Japan), while mycoplasma contamination was assessed with a MycoAlert detection assay (Lonza Rockland Inc., Rockland, ME, USA). HLA class II-restricted long peptides were introduced to the culture prior to the addition of maturation factors. The peptide-pulsed DCs were then suspended in 0.5 mL of saline within a 1 mL syringe and administered to patients by a trained physician. Injections were performed using a 25-G needle under ultrasound guidance, targeting the corticomedullary border of normal inguinal lymph nodes, as described previously [29].

### 2.6. Administration of Neo-P DC Vaccine Therapy

**Figure 1:**
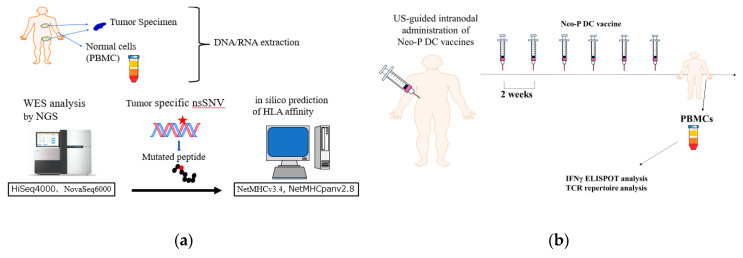
Protocol of intranodal neoantigen peptide-pulsed dendritic cell (Neo-P DC) vaccine therapy. (**a**) Tumour sampling (formalin-fixed paraffin-embedded tissue), genetic testing for neoantigen prediction using whole exome sequencing (WES) by next-generation sequencing (NGS) combined with in silico analysis, leukapheresis, and the synthesis of predicted neoantigens were performed. (**b**) For vaccine treatment, monocyte-derived DCs were cultured with neoantigen peptides and administered to patients via ultrasound (US)-guided intranodal injection. After the administration of six cycles of Neo-P DC at 2-week intervals, IFN-γ ELISpot analysis was performed. Peripheral blood mononuclear cell (PBMCs) and plasma obtained by leukapheresis and cryopreserved before and after treatment were used for analyses. T-cell receptor (TCR) analyses were performed for three patients.

Between November 2020 and November 2024, five patients received Neo-P DC vaccine therapy at the Department of Cancer Immunotherapy, Fukuoka General Cancer Clinic (Fukuoka, Japan) following surgery. The protocol for the Neo-P DC vaccine therapy is outlined in Figure 1. The vaccinations were administered via ultrasound-guided intranodal injections at two-week intervals. After completing six vaccinations, peripheral blood mononuclear cells (PBMCs) were isolated to evaluate immune responses to each neoantigen peptide using interferon-γ (IFN-γ)-based enzyme-linked immunospot (ELISpot) assays. PBMCs and plasma samples were collected at two stages: before treatment and after the completion of the six vaccinations.

### 2.7. IFN-γ ELISpot Assay

The ELISpot analysis was conducted using the Human IFN-γ ELISpot Plus Kit (Mabtech Inc., Cincinnati, OH, USA) in accordance with the manufacturer’s protocol. In brief, 96-well plates with nitrocellulose membranes (Millipore, Molshelm, France) were precoated with a primary anti-IFN-γ antibody and pretreated overnight at 4 °C with RPMI-1640 medium supplemented with 10% autologous serum. Following medium removal, 5 × 10^3^ autologous immature DCs were added to each well. Synthesized short peptides were introduced after DC maturation, while synthesized long peptides were added prior to maturation. After three washes with RPMI-1640 medium, 2 × 10⁵ autologous peripheral lymphocytes, isolated from cryopreserved PBMCs obtained pre- and post-vaccine therapy, were added to each well. The cells were then incubated for 48 h. Subsequently, the plates were washed three times with PBS, and a detection antibody (7-B6-1-biotin) dissolved in PBS containing 0.5% foetal bovine serum (PBS–0.5% FBS) at a concentration of 1 mg/mL was added (100 µL/well). The plates were incubated for 2 h at 15–25 °C. After another PBS wash, the secondary antibody (streptavidin-horseradish peroxidase) diluted in PBS–0.5% FBS at 1:1000 was added (100 µL/well), and the plates were incubated for 1 h at 15–25 °C. Following additional PBS washes, TMB substrate solution (MABTECH) was added (100 µL/well), and the plates were allowed to react for 10 min before the reaction was stopped with deionized water. The spots were visualized and analysed using the automated ELISpot Reader 08 Classic (AID GmbH, Strasberg, Germany). Spot intensity and size were multiplied to calculate ELISpot activity, and the values of all spots were summed and divided by 1000 to obtain the final activity value.

### 2.8. Isolation of CD8-Positive T Cells and CD4-Positive T Cells

CD8-positive and CD4-positive T cells were isolated from patient PBMCs using anti-human CD8 or anti-CD4 antibody-coated microbeads with the MACS Beads Cell Separation Kit (Miltenyi Biotech, Bergisch Gladbach, Germany), in accordance with the manufacturer’s protocol. The purity of the isolated CD8-positive and CD4-positive T cells was determined via flow cytometry and exceeded 97%.

### 2.9. T-Cell Receptor (TCR) Sequencing Analysis

TCR sequencing was performed using previously established methods [30]. Briefly, total RNA was extracted from 2 × 10⁶ T cells and utilized for cDNA synthesis with a universal 5′-RACE adapter, employing the SMART Library Construction Kit (Clontech Laboratories, Mountain View, CA, USA). PCR amplification was then conducted on TCRα and TCRβ cDNAs using a forward primer specific to the SMART adapter and a reverse primer targeting the constant region of the TCRα and TCRβ genes. Illumina index sequences with barcodes were incorporated using the Nextera XT Index Kit (Illumina, San Diego, CA, USA). The prepared libraries were sequenced on the Illumina MiSeq System with 300 bp paired-end reads, utilizing the MiSeq Reagent v3 600-cycle kit (Illumina). Sequence data analysis was performed with Bowtie2 aligner v2.5.0 [30]. TCR clonality was evaluated by calculating the inverse Simpson’s diversity index and the Shannon index based on CDR3 sequence information.

### 2.10. Statistical Analysis

Data are expressed as the mean ± standard deviation (SD). Statistical analyses and graph generation were performed using GraphPad Prism Version 8.3.0 (GraphPad software, La Jolla, CA, USA).

## 3. Results

### 3.1. Treatment with Neo-P DC Vaccine for Postoperative Breast Cancer Patients

Neo-P DC vaccines were administered to five post-operative breast cancer patients with the aim of preventing recurrence. Table 1 show the clinical characteristics of the patients in this study. One patient (BC3) had a recurrence of cervical lymph nodes, but it was only a local recurrence, so curative surgery (cervical lymph node dissection) was performed. The other four patients were diagnosed with early-stage breast cancer and underwent curative total mastectomy and sentinel lymph node biopsy. All patients underwent Neo-P DC vaccine as adjuvant settings along with standard treatment (Table 1). Three patients underwent standard adjuvant chemotherapy (anthracycline and taxane) before Neo-P DC vaccine treatment. Vaccine treatment was administered to all patients with the protocol shown in Figure 1. Table 3 shows the list of neoantigens selected.

### 3.2. Immune Responses After Intranodal Neo-P DC Vaccine Administration

**Figure 2:**
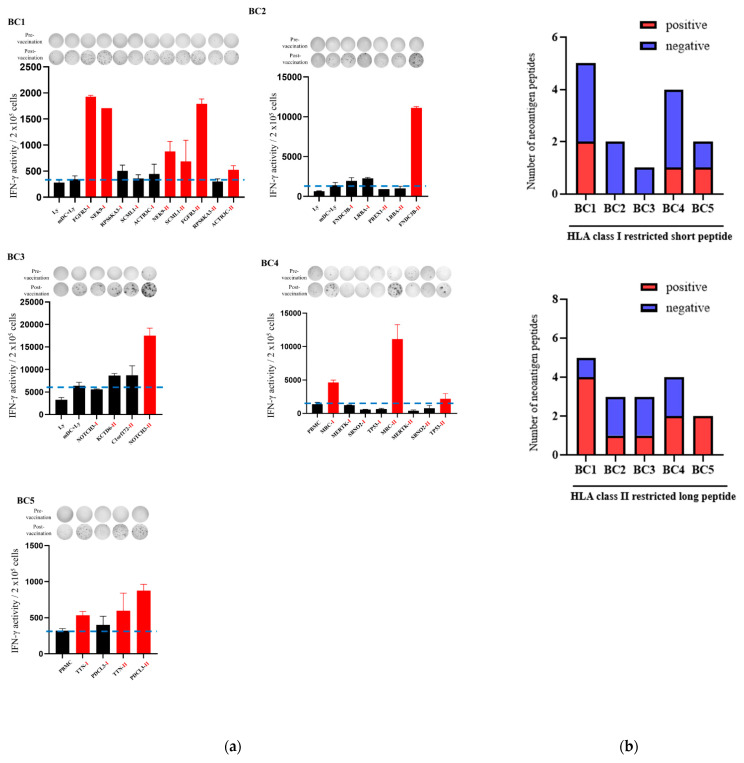
Immune responses of peripheral blood lymphocytes to neoantigen peptides after Neo-P DC vaccine treatment. (**a**) IFN-γ ELISpot responses to neoantigen peptides in peripheral blood lymphocytes from each patient after six cycles of vaccine treatment. The intensity and size of each spot were multiplied, and values of all spots were summed; the results were divided by 1000 to obtain the activity values. All measurements were performed in duplicate. Data are represented as mean ± SD. I and II indicate HLA class I or II neoantigen peptide derived from the mutated genes, respectively. Ly, lymphocytes; mDC+Ly, mature dendritic cells + lymphocytes; PBMC, peripheral blood mononuclear cell. The dotted blue horizontal line indicates the control level; the control is mDC + Ly in BC1, BC2, and BC3 and PBMC in BC4 and BC5. Each peptide is added to the control (PBMC or mDc+Ly). The red bars indicate positive activity. (**b**) Number of neoantigen peptides that evoked a positive or negative reaction in peripheral lymphocytes after Neo-P DC vaccine treatment.

Detailed immunological analysis was performed on all five patients treated with the HLA class I/II-restricted Neo-P DC vaccine. The results of IFN-γ ELISpot assays for each neoantigen peptide using peripheral blood lymphocytes isolated before and after the six vaccine doses are shown in Figure 2a. In two patients (BC4, BC5), the background IFN-γ production was remarkably enhanced after vaccination, so we used PBMCs, not mDC+ lymphocytes, as a control. We defined neoantigen peptides that evoked increases in the IFN-γ-producing activity of lymphocytes of 1.5 times or more compared with the control as positive and those that evoked activity less than 1.5 times as negative. The number of positive and negative neoantigen peptides in patients is shown in Figure 2b.

Although the degree of the enhancement of lymphocyte responses against neoantigens after Neo-P vaccine treatment differed among patients, enhanced lymphocyte IFN-γ production was detected against at least one HLA class II-restricted long peptide in all cases. Importantly, in BC1, BC3, and BC5, there was also an increase in the response to HLA class I-restricted peptides whose amino acid sequences are contained within the sequence of HLA class II-restricted peptides that evoked a positive reaction in peripheral lymphocytes.

### 3.3. HLA Class II-Restricted Neoantigen Long Peptide Encompassing HLA Class I-Restricted Epitope-Pulsed DCs Activated CD8-Positive T Cells and CD4-Positive T Cells

**Figure 3:**
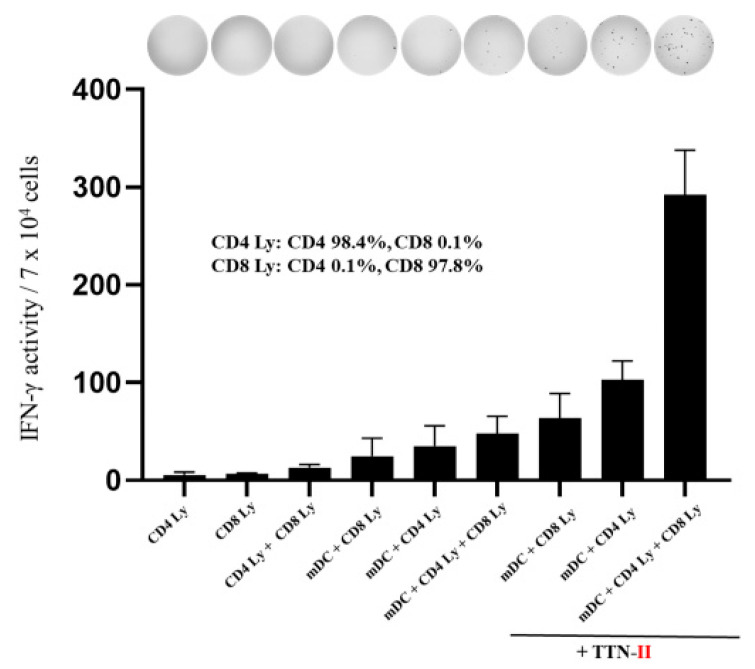
Immune responses of peripheral CD4-positive and CD8-positive T cells to neoantigen peptides after Neo-P DC vaccine treatment. IFN-γ ELISpot responses to the HLA class II-restricted mutant long peptide (TTN-II) in peripheral blood lymphocytes from each patient after six cycles of vaccine treatment. Spot activity values were calculated. All measurements were performed in triplicate. Data are represented as mean ± SD. In the evaluation of both CD4-positive and CD8-positive T cells, 3.5 × 10^4^ cells each were added to each well.

To investigate the mechanism by which HLA class II-restricted neoantigen long peptides encompassing HLA class I-restricted epitope-pulsed DC vaccines elicited peripheral lymphocyte reactivity to both HLA class I and II neoantigen peptides, we examined the responses of CD8-positive T cells and CD4-positive T cells restimulated by mDCs + HLA class II-restricted neoantigen long peptide in BC5. We selected the HLA class II-restricted neoantigen long peptide derived from the nonsynonymous SNV from TTN that included an amino acid sequence with high affinity to HLA class I. As shown in Figure 3, both CD8-positive T cells (CD8 purity = 97.8%) and CD4-positive T cells (CD4 purity = 98.4%) obtained after Neo-P DC vaccination responded to mDCs pulsed with the HLA class II-restricted mutant long peptide (TTN-II). Notably, when mDCs pulsed with HLA class II-restricted neoantigen peptides were co-cultured with CD4-positive T cells and CD8-positive T cells, the level of lymphocyte activation was enhanced beyond the sum of activation from their individual co-cultures.

### 3.4. TCR Repertoire Changes After Neo-P DC Vaccine

**Figure 4:**
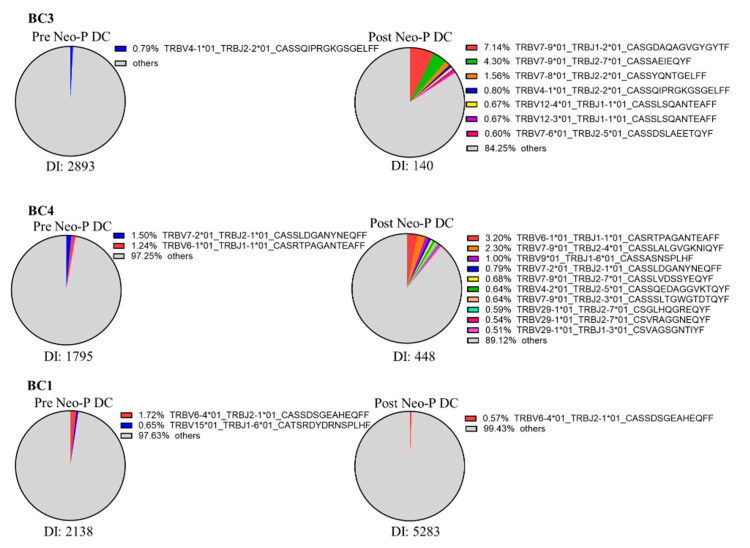
TCR repertoire analysis before and after Neo-P DC vaccine treatment. TCRβ repertoire analysis was performed in three patients from samples before and after vaccine treatment. CDR3β clonotypes accounting for more than 0.5% are shown in the pie chart. DI: diversity index.

We performed TCR repertoire analysis before and after Neo-P DC vaccine treatment for three patients (BC1, BC3, and BC4) (Figure 4). In BC3 and BC4, several TCRβ clonotypes (accounting for more than 0.5%) emerged after vaccination, and the diversity index decreased, suggesting that the Neo-P DC vaccine induced several antigen-specific T-cell activations. In BC1, there was no increase in a specific TCRβ clonotype.

## 4. Discussion

The dendritic cell vaccine therapy analysed in this study is a vaccine that uses the patient’s own monocyte-derived dendritic cells with the addition of cancer-specific antigen peptides and is a highly safe and scientifically rational cell therapy. We used the neoantigen prediction pipeline to predict HLA class I-restricted short peptides and HLA class II-restricted long peptides and then pulsed monocyte-derived dendritic cells with the synthetic neoantigen peptides. We then administered these to the inguinal lymph nodes of five breast cancer patients as a dendritic cell vaccine therapy with the aim of preventing recurrence after curative surgery. Retrospective analysis was conducted on the immune response after vaccine administration. The following results were obtained.

(1) The peripheral lymphocyte response was enhanced for both HLA class I-restricted neoantigens and HLA class II-restricted neoantigens. The increase in lymphocyte response was particularly marked for HLA class II-restricted neoantigens containing HLA class I affinity neoantigen epitopes.
(2) TCR repertoire analysis of three patients before and after vaccination showed that clonality increased in two of the three cases after Neo-P DC vaccination.
(3) At the time of publication, there have been no relapses or adverse events in the five breast cancer patients.

While clinical trials of neoantigen vaccine therapy for breast cancer have recently begun, so far, there has been no research published [31]. We have started neoantigen vaccine therapy as a cell therapy called cancer antigen-stimulated dendritic cell vaccines under the Act on the Safety of Regenerative Medicine in Japan and have published on its safety and immunological efficacy [20,29]. The current study is the first report on the immunological analysis of neoantigen vaccine therapy for the purpose of preventing recurrence in breast cancer.

Until now, most of the neoantigens used in clinical trials have been short peptides of 8–11 amino acid sequences that are recognized by CD8-positive T cells in a state bound to HLA class I molecules. These have been used as materials for neoantigen vaccines in the form of peptides or converted to mRNA. However, recent research has revealed the importance of HLA class II-restricted neoantigens that activate CD4-positive helper T cells. In this study, we predicted HLA class II-restricted neoantigen peptides and HLA class I-restricted neoantigen peptides, synthesized these as long peptides of 15–18 amino acid sequences, and used them as the source of the neoantigen-pulsed DC vaccine. Our research demonstrated that, following administration of this Neo-P DC vaccine, increased lymphocyte reaction against at least one HLA class II-restricted long peptide was detected, and the increase in response was equivalent to or greater than that of lymphocytes that react to HLA class I neoantigens. This result is consistent with another study that showed that CD4-positive T-cell responses were more strongly induced than CD8-positive T-cell responses when mRNA vaccines targeting both HLA class I-restricted neoantigens and HLA class II-restricted neoantigens were administered in melanoma patients [11].

Recent studies have revealed the importance of HLA class II-restricted neoantigens that activate CD4-positive helper T cells [14,15,32,33,34]. However, there have been no reports of dendritic cell vaccines that target both HLA class I- and class II-restricted neoantigens. The HLA class II-restricted neoantigen peptides we used contained HLA class I-restricted neoantigen epitopes, and it is possible that they activated both neoantigen-reactive CD4-positive helper T cells and CD8-positive T cells. Indeed, the IFN-γ ELISpot data for BC5 showed that when HLA class II-restricted neoantigen peptide-pulsed DCs and CD4-positive T cells and CD8-positive T cells obtained after Neo-P DCs vaccination were co-cultured, the degree of lymphocyte activation increased even further than the sum of activation levels when they were co-cultured separately. This suggested that HLA class II-restricted neoantigen-presenting dendritic cells that contain HLA class I-restricted neoepitopes may synergistically activate CD4-positive T cells and CD8-positive T cells.

The results of our study have suggested the usefulness of HLA class II-restricted neoantigen vaccine therapy in preventing breast cancer recurrence. Recent reports on neoantigen-based vaccine therapy have demonstrated significant effects in cases in which HLA class II-restricted neoantigen peptide-specific T cells were used [35,36]. Together, these results indicate the potential for the development of a treatment strategy that targets neoantigen-specific CD4 positive T cells.

To investigate whether the administration of neoantigen vaccines increase specific T-cell clones, we performed TCR repertoire analysis in three patients who had received Neo-P DC vaccines. In two cases (BC2, 3), several specific TCRβ clonotypes increased, while in the other case, there was no increase in specific TCRβ clonotype, and TCR diversity increased. In the two cases in which TCR repertoire clonality increased, the number of increased TCRβ clonotypes was larger than the number of neoantigens used in the vaccine. Because there was a marked increase in lymphocyte response to several HLA class II-restricted neoantigens, we speculate that strong antitumor immune effect may have occurred and induced antigen spreading in these two cases. However, further research is needed to confirm this possibility. In the third case (BC1), no specific TCRβ clonotype increased despite the observed responses of peripheral lymphocytes to neoantigen peptides after the administration of Neo-P DC vaccines. This may indicate that the proportion of lymphocytes specific to neoantigen vaccines do not necessarily increase in the peripheral blood even when they are activated. Indeed, one study reported that peripheral blood lymphocytes (PBLs) could exhibit antigen-specific reactivity without significant expansion unless conditions like targeted stimulation such as in vitro stimulation were met. This suggests that peripheral antigen-specific responses may underrepresent the actual clonal expansion occurring in localized tissues, such as tumours or lymphoid organs [37]. These findings indicate the need to validate the effectiveness of neoantigen vaccines through additional tests, such as in vitro stimulation of peripheral lymphocytes with the neoantigen.

The significance of the TCR repertoire in cancer vaccines is still controversial. One study reported that TCR diversity increased after the combined use of cancer peptide vaccines and chemotherapy, and this correlated with a good prognosis [38]. Another study showed that the changes in the TCR repertoire differ depending on the method of vaccine, such as the TCR changing to polyclonal in the case of peptide-pulsed dendritic cell vaccines and the TCR clone becoming monoclonal in the case of peptide vaccines alone [39]. Furthermore, in a clinical trial in which an immune checkpoint inhibitor was used in combination with a neoantigen vaccine, cases in which clonality in the TCR repertoire increased had a good prognosis [40]. These results suggest that the way in which neoantigen vaccines are administered and the presence or absence of combination therapies such as immune checkpoint inhibitors and chemotherapy may affect the changes in the TCR repertoire after vaccination. Thus, further investigation is needed to analyse the TCR repertoire after neoantigen vaccine treatment and its significance.

There are several methods of administering neoantigen-stimulated dendritic cells, including intradermal, intravenous, and lymph node administration. We used a lymph node administration method under ultrasound guidance, and the usefulness, safety, and chemical rationality of this method have been shown in a recent review [32]. We speculate that the direct injection of dendritic cells into the lymph nodes may have induced a high immune response by ensuring that neoantigen-reactive lymphocytes proliferated quickly.

Several limitations in this study should be stated. First, the number of specimens was small. Setting a longer study period would increase the sample size, but this was a pilot study and the research was aimed at future larger-scale clinical trials, so the results were reported at this stage with a small number of cases. It will be necessary to analyse more cases and investigate correlations with the number of neoantigens and the response of neoantigen-reactive T cells in future studies. Second, we did not examine tumour heterogeneity [41,42]. It is reported that tumours exhibit heterogeneity as a result of clonal evolution, meaning that the neoantigen profile of the entire tumour may not be fully captured by analysing neoantigens from just one section. Therefore, we are considering analysing multiple regions of the same tumour. Finally, this study did not confirm whether the T-cell clones that increased in peripheral blood following neoantigen vaccine administration were indeed neoantigen-specific or capable of attacking tumours. The post-treatment analysis was limited by the small quantity of samples obtained through peripheral vein blood sampling, which restricted the number of cells available for experiments, including ELISPOT assays and TCR repertoire analysis. More detailed analyses will be required, such as TCR tetramer analysis or the creation of organoids from tumour samples to perform cytotoxicity assays. These points are crucial for the practical application of neoantigen vaccines as a cancer treatment.

## 5. Conclusions

In this study, we administered an HLA class II-restricted neoantigen peptide dendritic cell vaccine to breast cancer patients with the aim of preventing recurrence. We demonstrated the immunological efficacy of this vaccine against breast cancer. Our analysis provides useful information for the development of vaccine therapy for the prevention of breast cancer recurrence.

## Figures and Tables

**Table 1:** Clinical characteristics of the breast cancer patients.

ID	Age/Sex	Subtype	Biopsy Site	Adjuvant Therapy	Metastasis	Stage	Years Since Diagnosis
BC1	51/F	Luminal	Breast	ET	none	I	0
BC2	57/F	Luminal	Breast	ET	none	I	0
BC3	63/F	Luminal	LN	ET, CT	LN	IV	6
BC4	35/F	TNBC	Breast	CT	LN	II	0
BC5	37/F	Luminal	Breast	CT	none	II	0

LN, lymph node; TNBC, triple negative breast cancer; ET, endocrine therapy; CT, chemotherapy.

**Table 2:** The number of mutations and predicted neoantigens in patients.

ID	No. of Exonic Mutations	No. of nsSNVs	No. of HLA Class I Neoantigens	No. of HLA Class II Neoantigens
BC1	21	12	56	758
BC2	25	13	71	868
BC3	62	41	208	1241
BC4	52	25	72	1145
BC5	337	22	53	191

nsSNVs, non-synonymous single nucleotide variants.

**Table 3:** HLA class II neoantigens and their encompassing HLA class I neoantigen used in this study.

ID	Gene	Exp. Level	AA Change	HLA Class II Neoantigen	Affinity (nm), HLA Type	HLA Class I Peptide	Affinity (nm), HLA Type
BC1	NEK9	37	D542V	VQCGC**V**GTFLLTQSGKV	169, DRB1:0405	VQCGC**V**GTF	33, HLA-B15:01
	SCML1	25	R199Q	TAKVLCYYID**Q**LKQGKCF	165, DRB1:0405	**Q**LKQGKCF	151, HLA-B15:01
	FGFR3	31	G380R	AGSVYAGILSY**R**VGFFLF	85, DRB1:0405	SY**R**VGFFLF	14, HLA-B24:02
	RPS6KA3	47	P342L	FSTIDWNKLYRREIH**L**PF	52, DRB1:0405	KLYRREIH**L**PF	35, HLA-B15:01
	ACTR3C	5	R32L	VLAKAASWTSRQVGE**L**TL	124, DRB1:0901	RQVGE**L**TL	169, HLA-B15:01
BC2	PREX1	49	H1429Y	VFY**Y**IEGSRQALKVIFYL	17, DRB1:1201	VANTNVFY**Y**	17, HLA-B35:01
	LRBA	3	I1375R L1376R	VMDNMVMACGG**RR**PLLSA	35, DRB1:1201	MVMACGG**RR**	116, HLA-A11:01
	FNDC3B	41	Y175C	QEIIPF**C**GMSTYITR	87, DRB1:1201	IPF**C**GMSTY	6, HLA-B35:01
BC3	NOTCH3	3895	N1588H	SVVMLEID**H**RLCLQS	5, DRB1:0301	VMLEID**H**RL	9, HLA-A02:01
	KCTD6	279	S151F	TKVH**F**LLEGISNYFTKW	16, DRB1:1501	LTITTKVH**F**	23, HLA-B58:01
	C1orf172	419	P203R			SL**R**STFASSPR	43, HLA-A33:03
BC5	MRC2	386	R1437W	TAALILY**W**RRQSIER	3, DRB1:1201	TAALILY**W**R	12, HLA-A33:03
	MERTK	30	S972L	RLVRNGVSWSH**L**SMLPLG	38, DRB1:1201	LVRNGVSWSH**L**	89, HLA-B07:02
	SBNO2	12	V416M	VLDLQNKLPLAR**M**VYASA	10, DRB1:1201	LPLAR**M**VYASA	176, HLA-B07:02
	TP53	29	Y126C	TCT**C**SPALNKMFCQLAKTCPV	57, DRB1:1201	TCT**C**SPAL	394, HLA-C03:03
	FAM178A	1	P552R			RTKSPPAAL	107, HLA-B07:02
BC5	PDCL3	45	D67N	EE**N**ERAIEMYRRRRLAEW	68, DRB1:1454	EE**N**ERAIEMY	89, HLA-B44:03
	TTN	3	A16911S	YQFRIFAENRYGQSF**S**L	285, DRB1:1454	AENRYGQSF**S**L	11, HLA-B40:01

Underlined sequence indicates HLA class I neoantigen; changed amino acid is shown in bold. Exp. level, expression level (mRNA read count); AA change, position of amino acid change; Affinity, affinity to HLA (nm). The red text indicates the mutated amino acids.

## Data Availability

This study does not have any datasets that can be made publicly available.

## Supplementary Materials

The following supporting information can be downloaded at: www.mdpi.com/article/10.3390/cancers16244204/s1, Table S1: Pathological characteristics of the breast cancer patients.
