# Supplementary material for: Retrospective Analysis of HLA Class II-Restricted Neoantigen Peptide-Pulsed Dendritic Cell Vaccine for Breast Cancer
Source: Cancers (Basel). 2024 Dec 17;16(24):4204. doi: 10.3390/cancers16244204 (PMC11674441; doi:10.3390/cancers16244204)
Supplement: Supplementary file 1 [file cancers-16-04204-s001.zip › cancers-3357153-supplementary.pptx]

## Slide 1
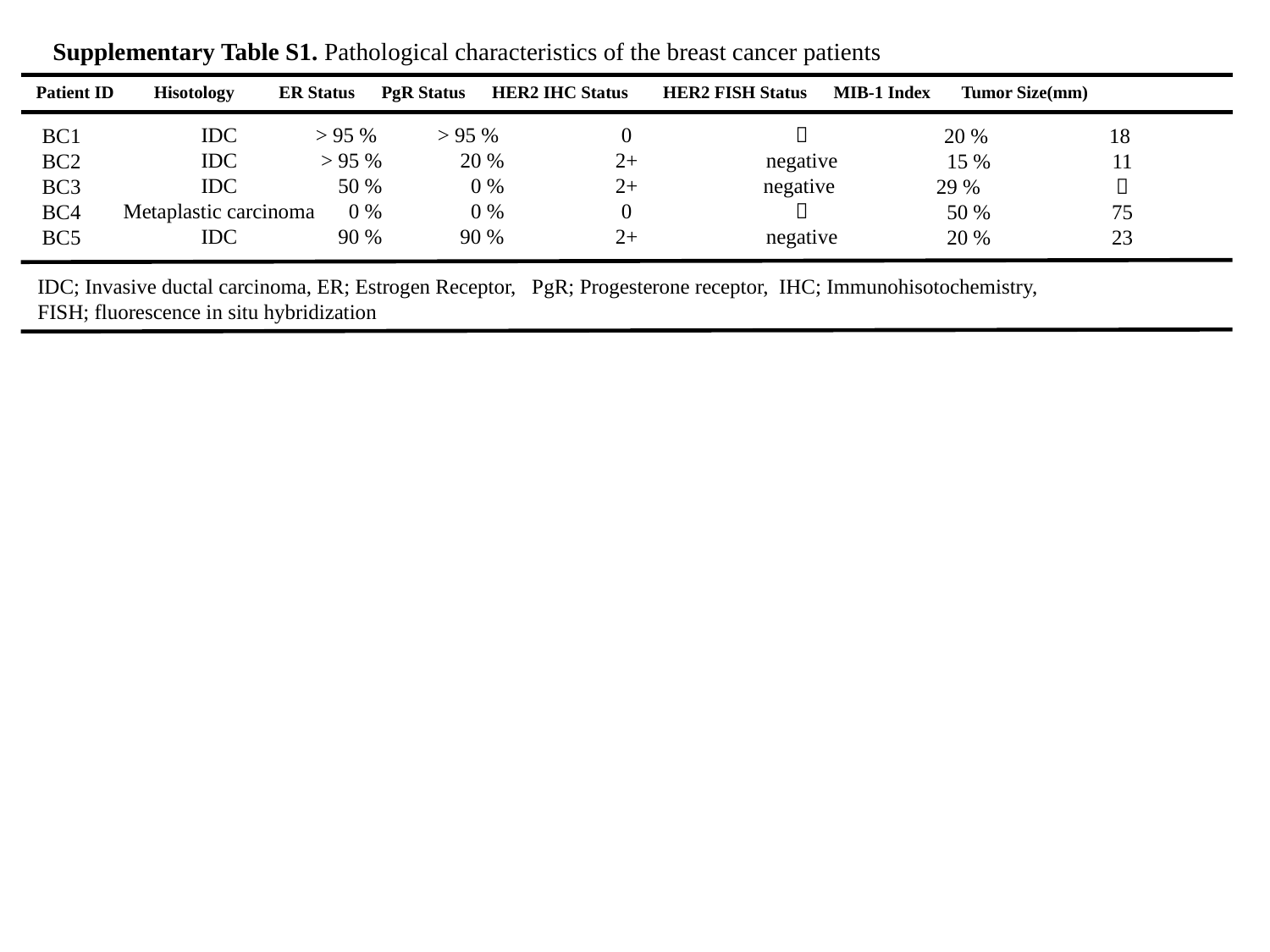

Supplementary Table S1. Pathological characteristics of the breast cancer patients
Patient ID Hisotology ER Status PgR Status HER2 IHC Status 　HER2 FISH Status MIB-1 Index Tumor Size(mm)
IDC
IDC
IDC
Metaplastic carcinoma
IDC
> 95 %
 > 95 %
50 %
0 %
90 %
> 95 %
 20 %
0 %
0 %
90 %
0
2+
2+
0
2+
ー
negative
negative
ー
negative
BC1
BC2
BC3
BC4
BC5
20 %
15 %
29 %
50 %
20 %
18
11
ー
75
23
IDC; Invasive ductal carcinoma, ER; Estrogen Receptor, PgR; Progesterone receptor, IHC; Immunohisotochemistry,
FISH; fluorescence in situ hybridization
